# Spaying the Female Canine

**Published:** 1880-07

**Authors:** 


### Art. XV.—SPAYING THE FEMALE CANINE.

### Directions for the Operation.

THE animal should not be less than four to six weeks, and not more than six months, old. The apartment in which the operation is to be performed should be previously heated up to a degree equal to the temperature of the animal's body (say, 98° to 100° F.), and maintained at this standard throughout. This is to prevent any shock which might occur were the vitals of the animal exposed to an atmosphere colder than its own body. Place the animal upon a table three feet in height, and bring it under the influence of an anesthetic; then empty the bladder by means of a catheter.

The abdominal cavity is now to be opened in the following manner: Make an incision of a sufficient length through the abdominal walls, in the *linea alba*, or median line, in the space contained between the *umbilicus* and the *symphysis pubis*. After the opening has been made, the ovaries will be found by following up the right and left branches of the *uterus*, or womb, to their outer extremities. The ovaries may be readily and easily recognized as firm nodular bodies, one on each side, about the size and shape of a flattened garden pea, or bean, according to the size and age of the animal to which they belong. To secure and remove these constitutes the operation of spaying. This may be accomplished either by pinching them off at the pedicle, between the finger and thumb nail, or by first placing around the pedicle a ligature of fine silk, and then cutting them off close to the ligature with a pair of scissors. The first method may be followed by some degree of hemorrhage; but, in the last, the tightly-drawn ligature prevents this. After the ovaries have been removed, the opening made into the abdominal cavity is to be closed by means of sutures of silk, placed half an inch apart—the ordinary interrupted suture—strips of adhesive plaster to be applied in the spaces between the sutures, in order to make the closure more complete, and everything more secure. This concludes the operation. Let the animal now be wrapped in a blanket, placed in a warm room, and there be allowed to remain in quietness.

When spaying is done in the manner here detailed, the operation usually results in success, but few animals dying from its effects. The median line is selected for the incision, because here it is not so likely to weaken the abdominal walls, as it must necessarily do when made after the usual method—through the flank.

Bitches, when spayed at or about the age specified, grow up to be beautiful animals, and make much more desirable house-pets or watch-dogs than either the male or the female in their natural state. When operated upon so young, they do not become fat and sluggish, but retain in general all their most useful qualities.

The above directions for spaying the female canine are essentially the same as those published by "South Fork" in *Forest and Stream* in 1877.

